# The variation of intestinal autochthonous bacteria in cultured tiger pufferfish *Takifugu rubripes*

**DOI:** 10.3389/fcimb.2022.1062512

**Published:** 2022-12-13

**Authors:** Lei Gao, Ziyang Zhang, Zhen Xing, Qingsong Li, Ning Kong, Lingling Wang, Linsheng Song

**Affiliations:** ^1^ Liaoning Key Laboratory of Marine Animal Immunology and Disease Control, Dalian Ocean University, Dalian, China; ^2^ Liaoning Key Laboratory of Marine Animal Immunology, Dalian Ocean University, Dalian, China; ^3^ Dalian Key Laboratory of Aquatic Animal Disease Prevention and Control, Dalian Ocean University, Dalian, China; ^4^ Laboratory of Marine Fisheries Science and Food Production Process, Qingdao National Laboratory for Marine Science and Technology, Qingdao, China

**Keywords:** pufferfish *Takifugu rubripes*, intestinal autochthonous bacteria, seawater bacteria, immune response, bacterial indicator

## Abstract

Intestinal autochthonous bacteria play important roles in the maintenance of the physiological homeostasis of animals, especially contributing to the host immune system. In the present study, the variation of autochthonous bacterial community in the intestinal tract of 2-7 months-old tiger pufferfish *Takifugu rubripes* and bacterial communities in the seawater of recirculating aquaculture system (RAS) and the following offshore sea cage aquaculture system (OSCS) were analyzed during the aquaculture period from May to October 2021. Proteobacteria was found to be the most dominant phyla in both intestinal and seawater bacterial communities, which accounted for 68.82% and 65.65% of the total bacterial abundance, respectively. *Arcobacter* was the most core bacterial taxon in the intestinal bacterial community, with the most dominant abundance (42.89%) at the genus level and dominant positions in co-occurrence relationships with other bacterial taxa (node-betweenness value of 150). Enterococcaceae was specifically enriched in the intestinal bacterial community of pufferfishes from RAS, while Vibrionaceae was enriched in the intestinal bacterial community from OSCS. The F-values of beta diversity analysis between intestinal and seawater bacterial communities generally increased from May (6.69) to October (32.32), indicating the increasing differences between the intestinal and seawater bacterial communities along with the aquaculture process. Four bacterial taxa of *Weissella* sp., *Akkermansia muciniphila*, *Dietzia* sp. and *Psychrobacter pacificensis* had significant correlations with immune response parameters, and they were suggested to be the indicators for immune status and pathological process of pufferfish. The knowledge about the specific core bacteria, potentially pathogenic bacteria and the change of bacterial community in the intestinal tract of cultured pufferfish is of great scientific significance and will contribute to the understanding of intestinal bacterial homeostasis and biosecurity practice in pufferfish aquaculture.

## Introduction

Tiger pufferfish (*Takifugu rubripes*) is one of model organisms, as well as a highly promising finfish species for aquaculture (Tao et al., 2012; Fishery Bureau of China Agriculture Department, 2021; Gao et al., 2022b), while the diseases of cultured pufferfish caused by intestinal microbiota disturbances and intestinal pathogens have been reported in recent years (Kodama et al., 2014; Sun et al., 2018; Li et al., 2019). The fish intestinal bacteria, especially autochthonous bacteria, contributes greatly to the immune response, development and growth of the hosts (Burns et al., 2016). The fish intestinal tract is therefore regarded as a critical immune organ and play important roles in the maintenance of physiological homeostasis and health of the hosts (Duan et al., 2020). The diversities, functions and symbiotic features of the intestinal bacteria have been investigated in some fish species, such as rainbow trout (*Oncorhynchus mykiss*), common carp (*Cyprinus carpio*) and turbot (*Scophthalmus maximus*) (Desai et al., 2012; Liu et al., 2019; Meng et al., 2021). The knowledge about the change of intestinal bacterial community during the aquaculture period of pufferfish will be crucial for the disease prevention and control.

Intestinal bacteria is constrained by many factors, such as environmental abiotic and biotic factors, host immune responses and nutritional status, among which environmental factors have a considerable impact on the structure and composition of the intestinal bacteria of fish through constant contact (Hopkins et al., 2002; Dethlefsen et al., 2006; Tanaka et al., 2009; Turnbaugh et al., 2009; Dehler et al., 2017; Kokou et al., 2019). There are two different aquaculture systems for pufferfish, named offshore sea cage aquaculture system (OSCS) and recirculating aquaculture system (RAS). The fingerlings are reared in RAS until around 10 g of body weight in late June and then transferred to OSCS. Fish are switched back to RAS in late October to overwinter and then transferred to OSCS again next May until harvest. The two aquaculture systems have different environmental factors, including environmental bacterial community, water temperature, dissolved oxygen and inorganic substance concentrations. The environmental factors in RAS are more restricted and stable than those in OSCS due to artificial manipulation. The pufferfishes in different aquaculture systems always display a wide variation in health status, survival rate and flesh quality (Jia et al., 2019), and it is partly due to the effect of environmental factors of different aquaculture systems on intestinal bacteria and physiological metabolism of pufferfish.

The intestinal bacteria is always viewed as an “extra organ” due to its important role in intestinal protection and homeostasis (Dehler et al., 2017). Intestinal bacteria not only hinders pathogens to colonize in the intestinal tract through antibiosis or niche exclusion, but also provides protection for host health by stimulating the immune response of mammals (Webb et al., 2016). Recently, the fish intestinal bacteria was also reported to form a complex and dynamic microbial ecosystem, which contributed to immune system priming, disease prevention and host nutrient acquisition (Gómez and Balcázar, 2008; Banerjee and Ray, 2017). Besides the variations of bacterial communities in healthy fish (de Bruijn et al., 2018), the effect of environmental factors on intestinal bacteria (Wang et al., 2018) and interactions between intestinal bacteria and immune response (Gómez and Balcázar, 2008; Nie et al., 2017) were also drew much interest recently. The immune system impacts the ecology of commensal bacterial communities, and the bacteria were also found to be able to elicit a mild inflammatory reaction and activate the innate immune response after they were colonized in zebrafish larvae (Montalban-Arques et al., 2015), and induce oxidative stress (Yin et al., 2015; Bandeira Junior and Baldisserotto, 2021). Some intestinal bacterial taxa were also reported as health indicators in marine invertebrate species (Xiong et al., 2015; Huang et al., 2018). However, the variation of the bacterial community of intestinal tract in pufferfish under different culture systems and the relationship between the intestinal bacteria and immune response are still not clear.

In this study, the characteristics of both intestinal and environmental bacterial communities were analyzed using high-throughput sequencing technologies with the main objectives to (1) understand the change of autochthonous bacterial community in the intestinal tract of cultured pufferfish, (2) compare the characteristics of the autochthonous bacterial communities in the in intestinal tract of pufferfish from RAS and OSCS systems, (3) explore the relationship between the intestinal bacterial community and the parameters involved in immune response including oxidative stress parameters as well as cytokines, which are secreted by the cells of immune system in immune responses and act as the means of communication (Stow et al., 2009). It is hopefully to identify the indicators of bacterial taxa for immune status and pathological process of pufferfish.

## Materials and methods

### Sample collection

The samples were collected in Dalian, China, from RAS system in May and June 2021, and OSCS system from July to October 2021. The geographic coordinates of RAS and OSCS are N39°36’20”–E122°51’12” and N39°28’12”–E123°4’59”, respectively. The samples were collected twice a month from the two systems at the same time interval, and nine fish were collected each time. The fish were anesthetized and dissected. The digestive tracts were excised, and the intestinal contents were squeezed out. The intestinal tract were rinsed three times with peptone water (1g/L bacterial peptone and 8.5 g/L NaCl) to remove the non-adherent bacteria and capture adherent bacteria (Ringø, 1993). Then, 0.1 g of the treated intestinal tract was taken from one individual, and the intestinal tracts from three individuals were pooled as one sample. For spleen samples, 0.1 g spleen was collected from one individual, and the spleens from three individuals were pooled as one sample. There were three replicates of spleen and intestinal tract samples for each sampling time, and they were stored at -80°C for further processing. Three-liter seawater (1 L for one sample) was collected at a depth of 1 m and then stored at 4°C for the analyses of water quality. Other 4 L seawater (1 L for one sample) was filtered using 0.22 μm pore size membranes (Sagon, Shanghai, China) to enrich the bacteria cells and stored at -80°C for DNA extraction.

### High−throughput sequencing and data processing

The bacteria genomic DNA from pufferfish intestinal tract and seawater were extracted by the Soil DNA Kit (Omega, Norcross, GA, USA) and Water DNA Kit (Omega, Norcross, GA, USA) respectively. DNA quality and quantity were analyzed with 1% agarose gel electrophoresis and NanoDrop spectrophotometer (Thermo Fisher Scientific, USA), respectively. The V3–V4 region of 16S rDNA genes was amplified using the primers of 515F (GTGBCAGCMGCCGCGGTAA) and 806R (GGACTACHVGGGTWTCTAAT). The high-throughput sequencing of V3–V4 region of 16S rDNA gene was performed on the Illumina HiSeq platform by Novogene (Beijing, China). The raw reads were trimmed and denoised by Quantitative Insights into Microbial Ecology 2 (QIIME 2) using “dada2 denoise-single” with default parameters and “–p-trim-left 0” and “–p-trunc-len 250”, to remove singletons and chimeric sequences and obtain amplicon sequence variants (ASVs) (Bolyen et al., 2019). Handled by QIIME 2 pipeline with the clean reads, taxonomy was assigned at a single nucleotide level with a feature classifier against the SILVA 138 SSU database, and alpha and beta diversity were analyzed by Chao1 index and unweighted UniFrac distances, respectively.

### Bioinformatics analysis

MicrobiomeAnalyst (http://www.microbiomeanalyst.ca) (Chong et al., 2020) was used to analyze the abundance and diversity of the bacterial community and generate visual exploration, and to conduct linear discriminant analysis effect size (LEfSe) analysis and identify the significantly differentially abundant taxa between intestinal and seawater bacterial communities, with *P*-value cutoff of 0.1 and Log LDA score of 2.0. The functional profiles and metabolic pathways were analyzed and predicted with Phylogenetic Investigation of Communities by Reconstruction of Unobserved States (PICRUSt) 2 software with the KEGG database for annotation (Douglas et al., 2020). STAMP software (version 2.1.3) was used for functional profile analysis (Parks et al., 2014). The co-occurrence ecological network was built using the online Molecular Ecological Network Analysis (MENA) pipeline (http://ieg2.ou.edu/MENA) (Deng et al., 2012), and the network construction was conducted using Cytoscape software (version 3.7.2) (Shannon et al., 2003).

### Determination of water quality indexes

Water temperature, dissolved oxygen (DO), pH and salinity were monitored by a YSI Professional Plus meter (YSI, Yellow Springs, Ohio, USA) *in situ*. The concentrations of TAN, NO_2_–N, NO_3_–N and PO_4_-P of the seawater samples were analyzed using the indophenol blue spectrophotometric method, N-(1-naphthyl)-ethylenediamine dihydrochloride spectrophotometric method, Zn-Cd reduction method and Phosphomolybdenum blue spectrophotometric method, respectively, according to the procedures of the National Specification for Marine Monitoring (SOA of China, 2007).

### Immune response parameter analysis

The activities of superoxide dismutase (SOD) in spleens were analyzed by water-soluble tetrazolium salt (WST-1) assay with assay kit (Jiancheng, Nanjing, China; Product code: A001-3-2). The contents of malondialdehyde (MDA) in spleens were analyzed by 2-thiobarbituric acid (TBA) method with assay kit (Jiancheng, Nanjing, China; Product code: A003-1-2). The experiments were carried out according to the manufacturer’s introductions. The SOD activity unit was defined as the amount of enzyme that can convert 1 μmol substrate. The total RNA was extracted from the spleen samples using TRIzol™ Reagent. RNA concentration was measured using a NanoDrop spectrophotometer, and the purity and integrity were examined through electrophoresis analysis. The first strand of cDNA was synthesized using the total mRNA as a template and oligo (dT)17-adaptor as a primer by PrimeScript^™^ RT Reagent Kit with gDNA Eraser (TaKaRa, Dalian, China) according to the manufacturer’s instruction. The synthesis reaction was conducted at 42°C for 5 min and terminated by heating at 85°C for 5 s. Temporal expression of IL-1β, TNF-α, IL10 and IL17-AF mRNA were measured by quantitative real-time PCR (qRT-PCR) using SYBR Green Master Mix (TaKaRa, Japan). The primer information was listed in **Table 1**. The expression of β-actin mRNA was used as the internal control. All reactions were performed in an ABI PRISM 7500 Detection System (Applied Biosystems, USA). The mRNA expressions were analyzed by the 2^-ΔΔCT^ method (Livak and Schmittgen, 2001).

**Table 1:** The primers used in this study.

Name	Sequence(5'-3')
β-actin-F	CAGGGAGAAGATGACCCAGA
β-actin-R	CATCACCAGAGTCCATGACG
IL-1β-F	TGAACCTGTCGACCTACGTG
IL-1β-R	ATACCAGGGTGCAGAGGTTG
TNF-α-F	AAGCTGCTACAACGCCATTT
TNF-α-R	TGATCTTCATGACCGTTGGA
IL10-F	CTTCTGGACCAAAGCATCGT
IL10-R	GACCTCGATCTTGAGCTGGT
IL17-AF-F	GTACGACTCACTATAGGGA
IL17-AF-R	AGGTGACACTATAGAATA

### Statistical analysis

The statistical analysis was conducted using one-way ANOVA by Statistical Package for Social Sciences (SPSS, version 26.0) (Field, 2013). The ASVs that were available in more than half of samples were screened for the correlation analysis with immune response parameters. The correlations between the abundance of bacterial taxa and immune response parameters were investigated through Spearman’s and Pearson’s correlation analysis using SPSS 26. The data were presented as means ± standard deviations with three parallel replicates. *P*-value < 0.05 was considered statistically significant.

## Results

### Composition and co-occurrence of intestinal bacterial community

**Figure 1:**
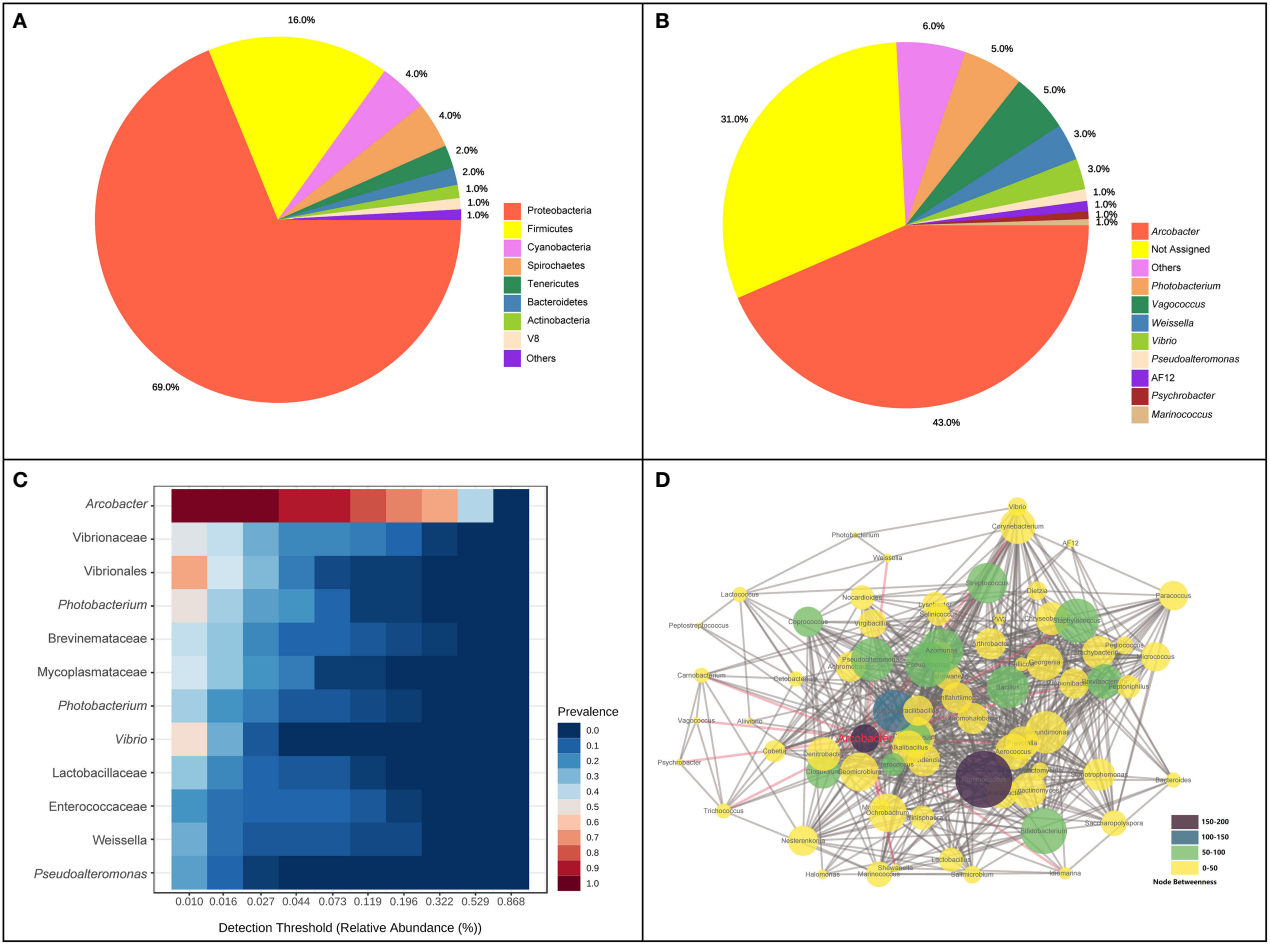
Composition and co-occurrence of intestinal bacterial community of pufferfish using all samples in this study. **(A)** The proportion of intestinal bacterial abundance at phylum level; **(B)** The proportion of intestinal bacterial abundance at genus level; **(C)** Core bacterial taxa in the intestinal bacterial community; **(D)** Microbial co-occurrence networks at the genus level.

A total of 2,865,508 reads were obtained after the high−throughput sequencing of intestinal tract samples. The mean read depth per sample was 79,597.4 sequences. The average length of the sequences was 253 bp. Across all samples, a total of 2,610 ASVs were detected in initial identification, with 1,979,446 appearance frequency in all samples. In total, 16 phyla were observed, and eight phyla were identified with abundance > 1%. Proteobacteria (68.82%), Firmicutes (16.09%), Cyanobacteria (4.35%), Spirochaetes (4.12%) and Tenericutes (2.01%) were dominant phyla in the intestinal bacterial community, accounting for 95.39% of the total abundance (**Figure 1A**). At the genus level, the five top dominant taxa were *Arcobacter* (42.89%), *Photobacterium* (5.33%), *Vagococcus* (5.16%), *Weissella* (3.21%) and *Vibrio* (2.7%), accounting for around 60% of the total abundance, and 31.27% of ASVs were failed to be annotated at the genus level. *Arcobacter* was found to be the most core bacterial taxon in the intestinal bacterial community of pufferfish (**Figure 1B**). When the detection threshold was set at 0.01%, the prevalence of *Arcobacter* was 97.2%; when the detection threshold was set at 0.321%, the prevalence of *Arcobacter* was still above 50% (**Figure 1C**). The co-occurrence analysis was conducted to investigate the interaction relationships among bacterial taxa and construct the microbial co-occurrence networks (**Figure 1D**). Among all genera identified in this analysis, *Ruminococcus* processed the dominant position in the co-occurrence relationships with the node-degree value of 51. *Ruminococcus* and *Arcobacter* were the top two betweenness hubs with the node-betweenness values of 190 and 150, respectively.

### The difference of intestinal bacterial communities in pufferfishes from RAS and OSCS systems

**Figure 2:**
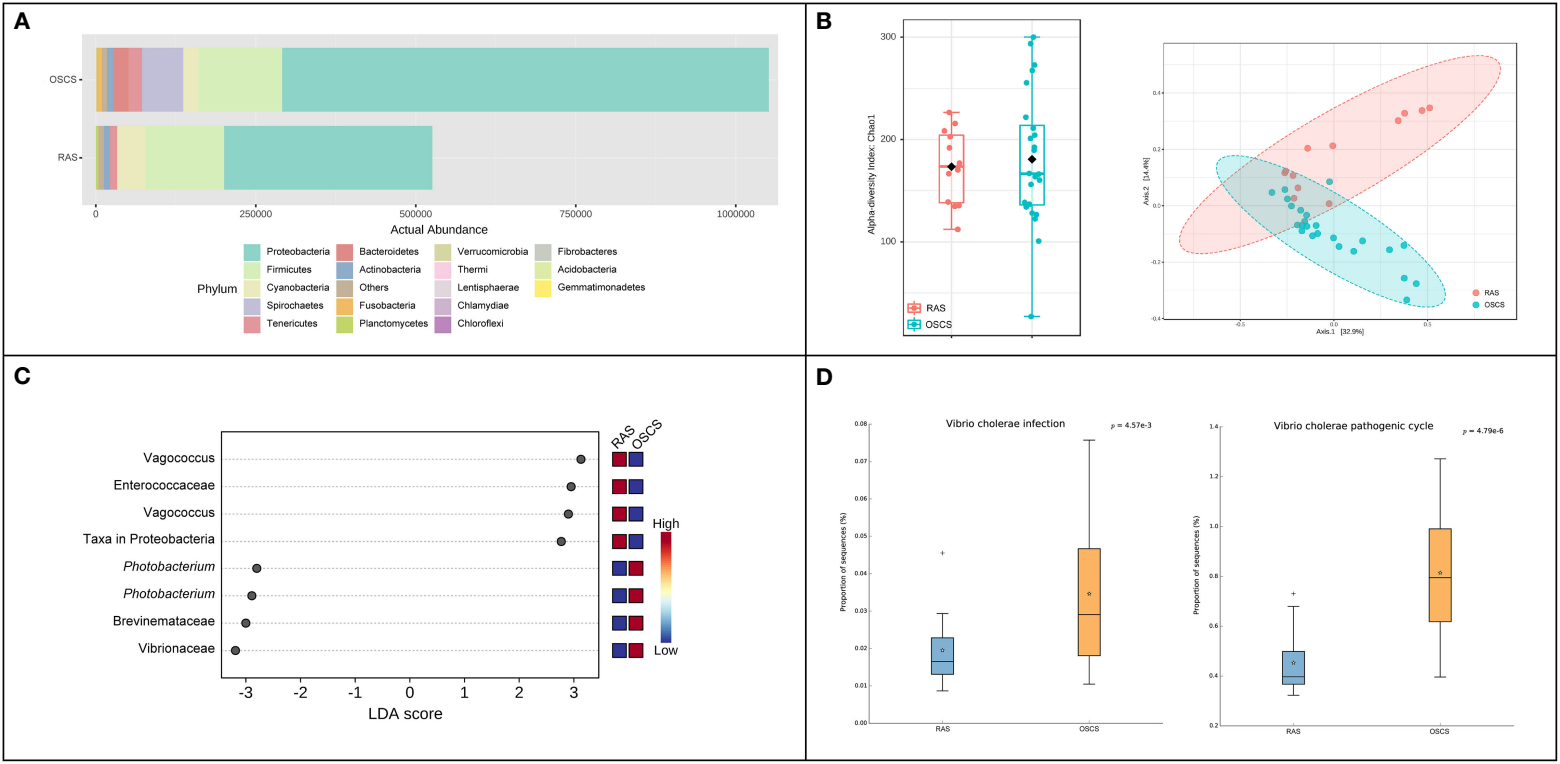
Intestinal bacterial communities in RAS and OSCS. **(A)** Bacterial abundance at phylum level; **(B)** LEfSe analysis of ASVs in intestinal bacterial communities between RAS and OSCS samples; **(C)** Alpha and beta diversity analyses of intestinal bacterial communities between RAS and OSCS samples; **(D)** Differences of functional pathways of intestinal bacterial communities between RAS and OSCS samples.

The abundance of the intestinal bacterial community in OSCS was about two-fold higher than that in RAS. At the phylum level, the relative ASV abundance of Spirochaetes and Bacteroidetes in OSCS (6.026% and 2.400%, respectively) were obviously higher than that in RAS (0.096% and 0.226%, respectively) (**Figure 2A**). The alpha diversity range of the intestinal bacterial community in OSCS was more variable than that in RAS. The result of beta diversity analysis showed that there was a distinct separation of intestinal bacterial communities between RAS and OSCS samples (**Figure 2B**). In LEfSe analysis, significant enrichments of ASVs were observed. The top-4 enriched ASVs in OSCS were Vibrionaceae, Brevinemataceae, *Photobacterium* and *Photobacterium*, with the LDA score of -3.19, -3.0, -2.9 and -2.8, respectively. The top-4 enriched ASVs in RAS were *Vagococcus*, Enterococcaceae, *Vagococcus* and Proteobacteria, with the LDA score of 3.13, 2.95, 2.9 and 2.77, respectively (**Figure 2C**). The functional profiles and metabolic pathways of intestinal bacterial communities were analyzed and predicted, and the functional pathways of *Vibrio* cholerae infection and *Vibrio* cholerae pathogenic cycle were found to be significantly more enriched in the intestinal bacteria community in OSCS than that in RAS (**Figure 2D**).

### Changes of water quality indexes in RAS and OSCS systems

**Figure 3:**
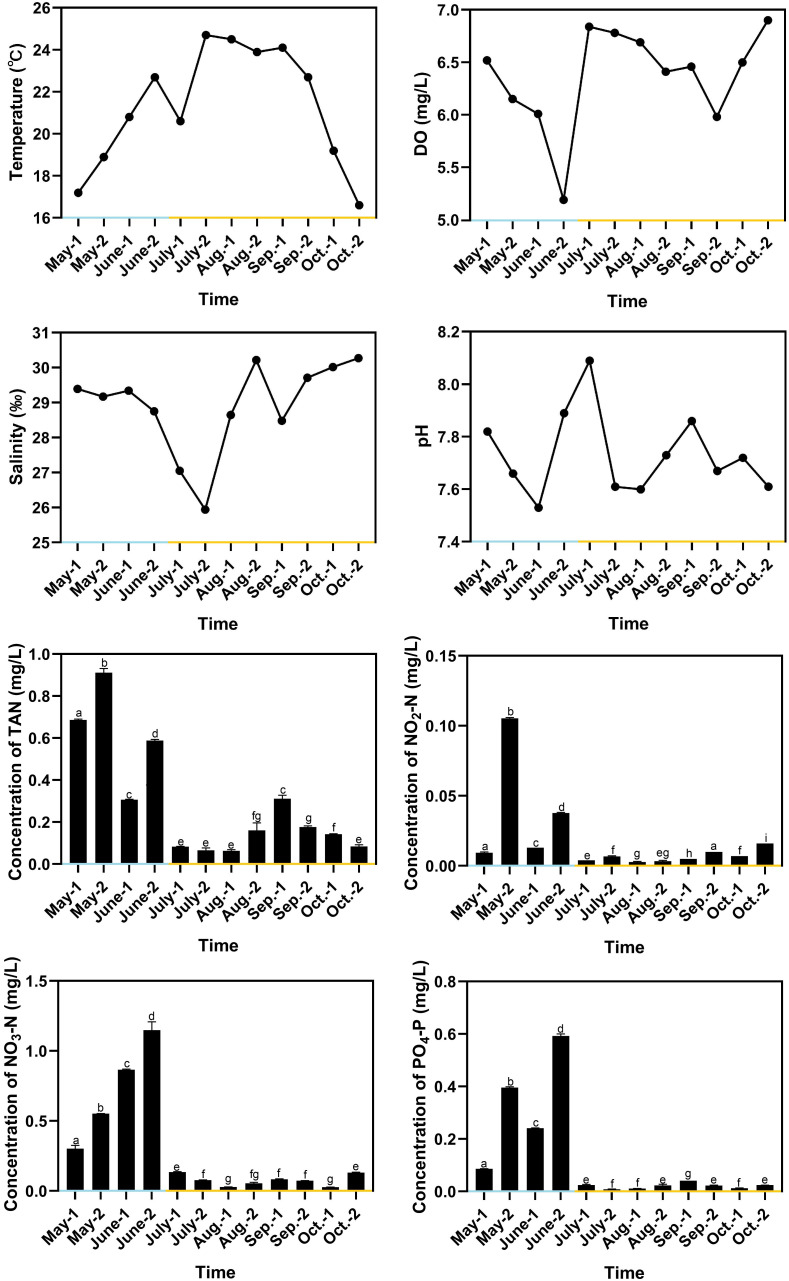
Variation of water quality indexes during aquaculture period. Blue x-axis indicates RAS aquaculture period, and yellow x-axis indicates OSCS aquaculture period. The letters above the bars indicate the significance of differences between groups; the groups with different letters have significant differences (P < 0.05).

The water temperature in RAS ranged from 17.2°C to 22.7°C, with the average value of 19.9°C, and the water temperature in OSCS ranged from 16.6°C to 24.7°C, with the average value of 22.0°C (**Figure 3**). The water temperature decreased to 20.6°C in early July due to rainfalls. pH in RAS ranged from 7.53 to 7.89, with the average value of 7.73, and pH in OSCS ranged from 7.60 to 8.09, with the average value of 7.74. The DO concentration varied and remained above 5 mg/L from May to October. The salinity ranged from 25.94 to 30.27 and reached the lowest value in late July due to rainfalls. The concentrations of TAN, NO_2_-N, NO_3_-N and PO_4_-P were generally higher in RAS than those in OSCS, with the highest values of 0.911, 0.105, 1.148 and 0.592 mg/L in RAS, respectively (**Figure 3**). The concentrations of TAN, NO_2_-N, NO_3_-N and PO_4_-P were found to have significant correlations with the aquaculture system (RAS/OSCS) (*P* < 0.05), with the correlation values of -0.81, -0.63, -0.83 and -0.81, respectively (**Table S1**). The concentration of PO_4_-P had significant correlations with the concentrations of TAN, NO_2_-N and NO_3_-N (*P* < 0.05), with the correlation values of 0.78, 0.67 and 0.91, respectively. The concentration of TAN had significant correlations with the concentrations of NO_2_-N and NO_3_-N (*P* < 0.05), with the correlation values of 0.70 and 0.61, respectively. The correlation analysis between water quality index and the beta diversity of intestinal bacterial community was conducted, and no significant correlation (*P* < 0.05) in both Spearman’s correlation analysis and Pearson’s correlation analysis was found (**Table S2**).

### Comparison between intestinal and seawater bacterial communities during the aquaculture period

**Figure 4:**
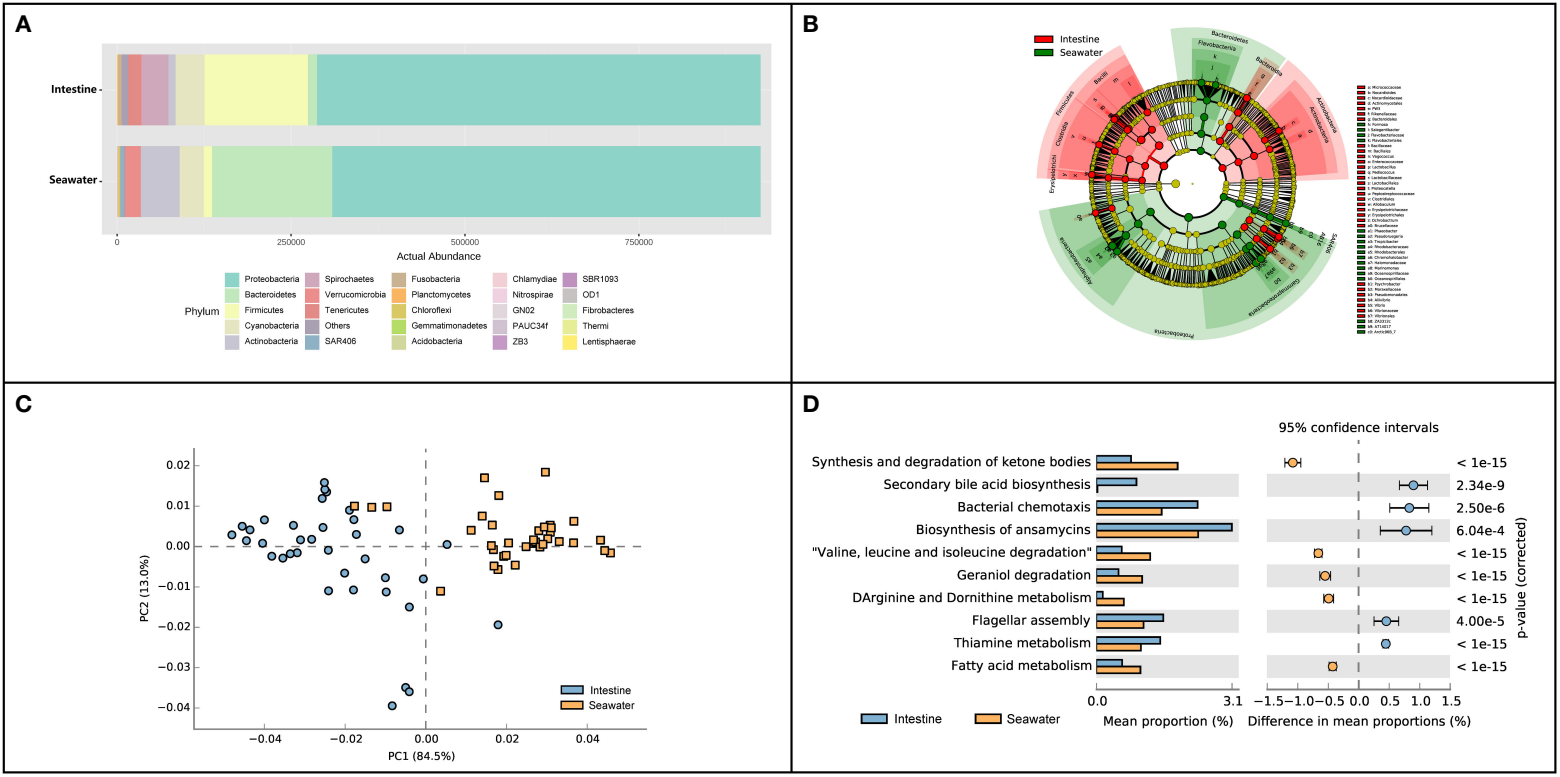
Composition and function differences between intestinal and seawater bacterial communities. **(A)** Bacterial abundance at the phylum level; **(B)** LEfSe analysis of ASVs between intestinal and seawater bacterial communities; **(C)** Principal components analysis (PCA) of bacterial functional characterization; **(D)** The differences of functional pathways between intestinal and seawater bacterial communities.

**Figure 5:**
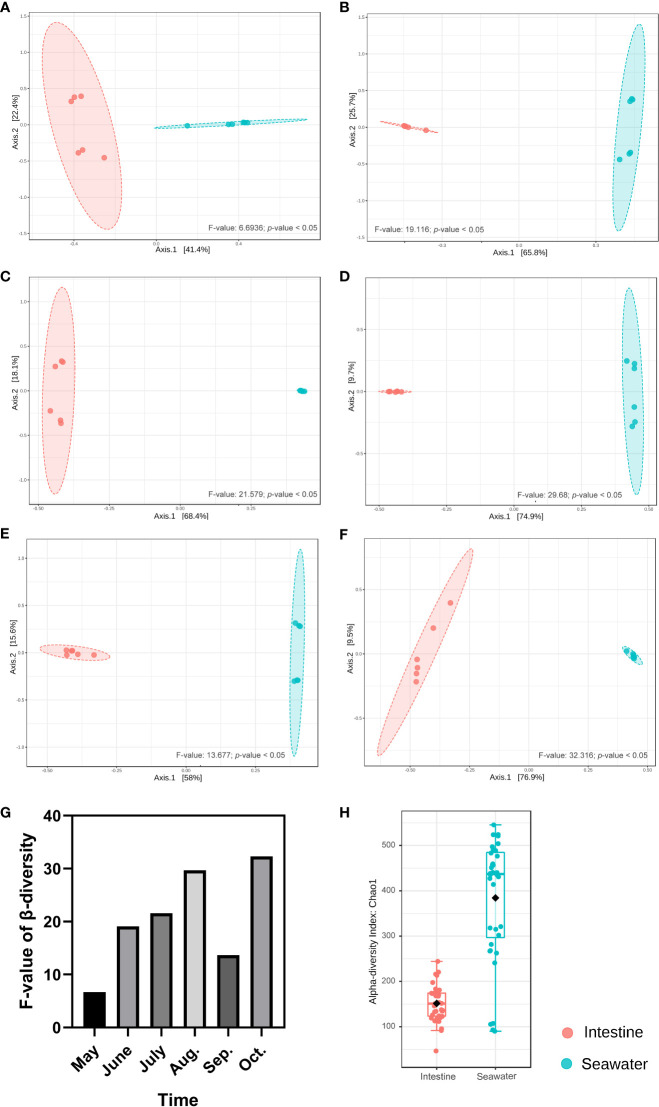
Beta and alpha diversity analyses between intestinal and seawater bacterial communities. **(A-F)** Beta diversity analyses of samples from May to October; **(G)** The F-values of beta diversity analyses in different months; **(H)** Alpha diversity analysis between intestinal and seawater bacterial communities.

In seawater, a total of 27 identified phyla were observed, and six phyla were identified with abundance > 1%. Proteobacteria (65.65%), Bacteroidetes (19.18%), Actinobacteria (6.02%), Cyanobacteria (3.85%) and Verrucomicrobia (2.46%) were dominant phyla in seawater bacterial community, accounting for 95.39% of the total abundance (**Figure 4A**). The correlation analysis of the abundance of the bacterial phyla that shared by intestinal tract and seawater samples was conducted, and it was found that the correlation values ranged from -0.323 to 0.188, and no significant correlation was observed (**Table S3**). There were noticeable differences in some phyla between the intestinal tract and seawater bacterial communities, including Bacteroidetes, Firmicutes, Actinobacteria, Spirochaetes, etc. According to LEfSe analysis, several bacterial taxa were identified to have significant differences between the intestinal tract and seawater bacterial communities, including Firmicutes, Bacteroidetes, Actinobacteria, SAR406 and Proteobacteria (**Figure 4B**). The PCA analysis of bacterial function showed clearly separate clustering of microbial function for intestinal and seawater bacterial communities (**Figure 4C**). There were significant differences in a lot of microbial functions, with the most prominent functions of synthesis and degradation of ketone bodies, secondary bile acid biosynthesis, bacterial chemotaxis, biosynthesis of ansamycins and valine, leucine and isoleucine degradation, etc. (**Figure 4D**). The F-values of beta diversity analyses generally increased with the sampling months, with the lowest value of 6.69 in May and the highest value of 32.32 in October (**Figures 5A-G**). The alpha diversity of the seawater bacterial community was significantly higher than that of the intestinal bacterial community (**Figure 5H**).

### Changes of immune response parameters

**Figure 6:**
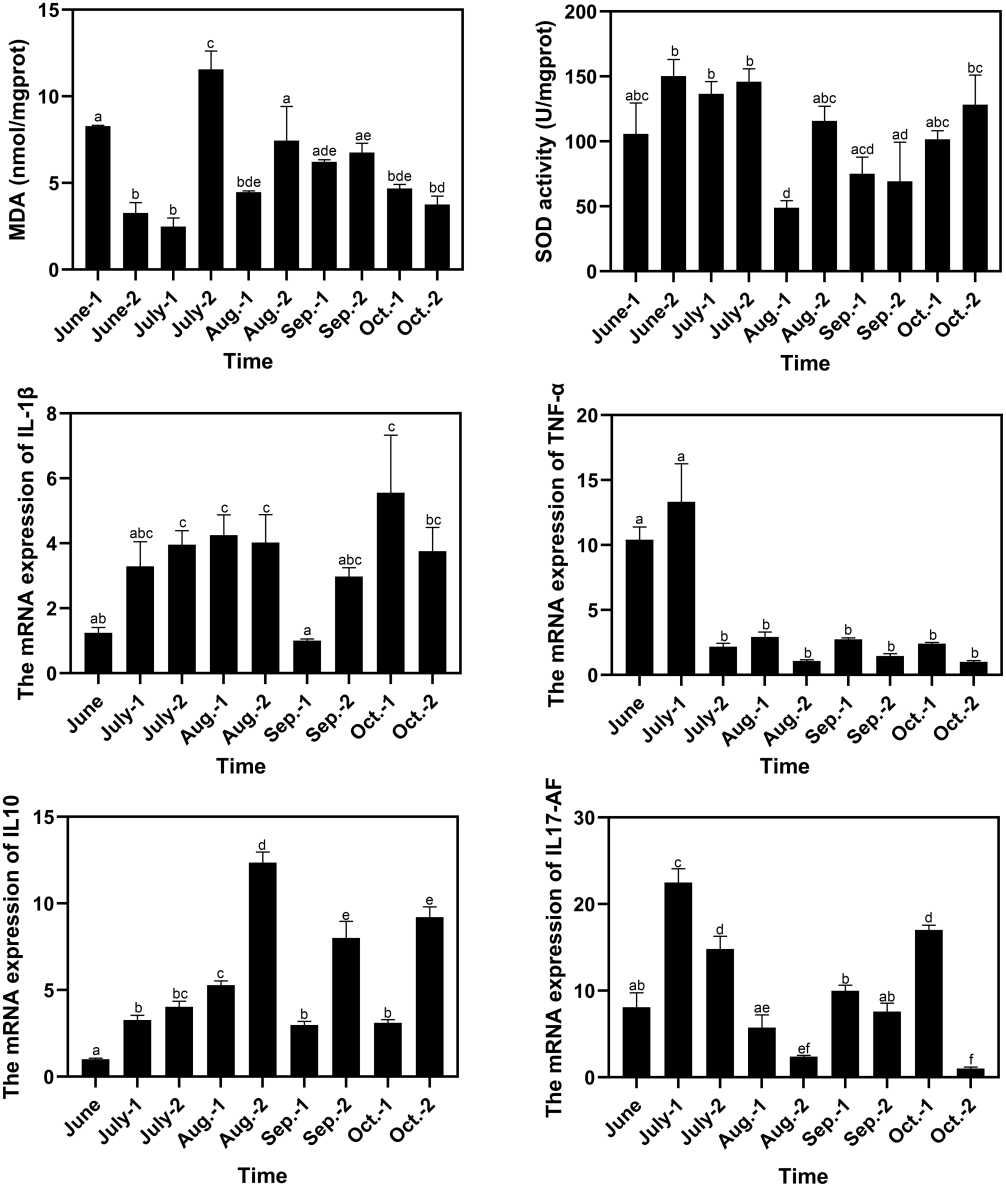
Variation of immune response parameters of pufferfish during the aquaculture period. The letters above the bars indicate the significance of differences between groups; the groups with different letters have significant differences (P < 0.05).

The MDA contents in spleen reached a peak of 11.55 nmol/mgprot in late July, which was significantly higher than those in the other sampling time points (**Figure 6**). The SOD activities decreased in early August to 48.98 U/mgprot, which was lower than those in most of the other sampling time points. The minimum and maximum values of the relative expression levels of IL-1β were observed in early September and early October, which were 0.81-fold and 4.47-fold of that in June, respectively. The relative expression levels of IL-1β significantly decreased in early September to 0.25-fold (*P* < 0.05) and 0.34-fold of that in late August and late September respectively. The minimum and maximum values of the relative expression levels of TNF-α were observed in late October and early July, which were 0.097-fold and 1.28-fold of that in June, respectively. There was a drastic drop in the relative expression levels of TNF-α in late July, and the low levels were maintained until the end of the experiment. The minimum and maximum values of the relative expression levels of IL10 were observed in June and late August, respectively, and the value in late August was 12.30-fold of that in June and was significantly higher than those in the other sampling time points. The minimum and maximum values of the relative expression levels of IL17-AF were observed in late October and early July, which were 0.13-fold and 2.78-fold of that in June, respectively. The relative expression levels of IL17-AF decreased gradually during July and August, with an increase from September to early October and a decrease in late October (**Figure 6**). The correlation analysis between water quality index and immune response parameter was conducted, and no significant correlation (*P* < 0.05) in both Spearman’s correlation analysis and Pearson’s correlation analysis was found (**Table S4**).

### Correlation analysis of intestinal bacterial taxa and immune response parameters

**Figure 7:**
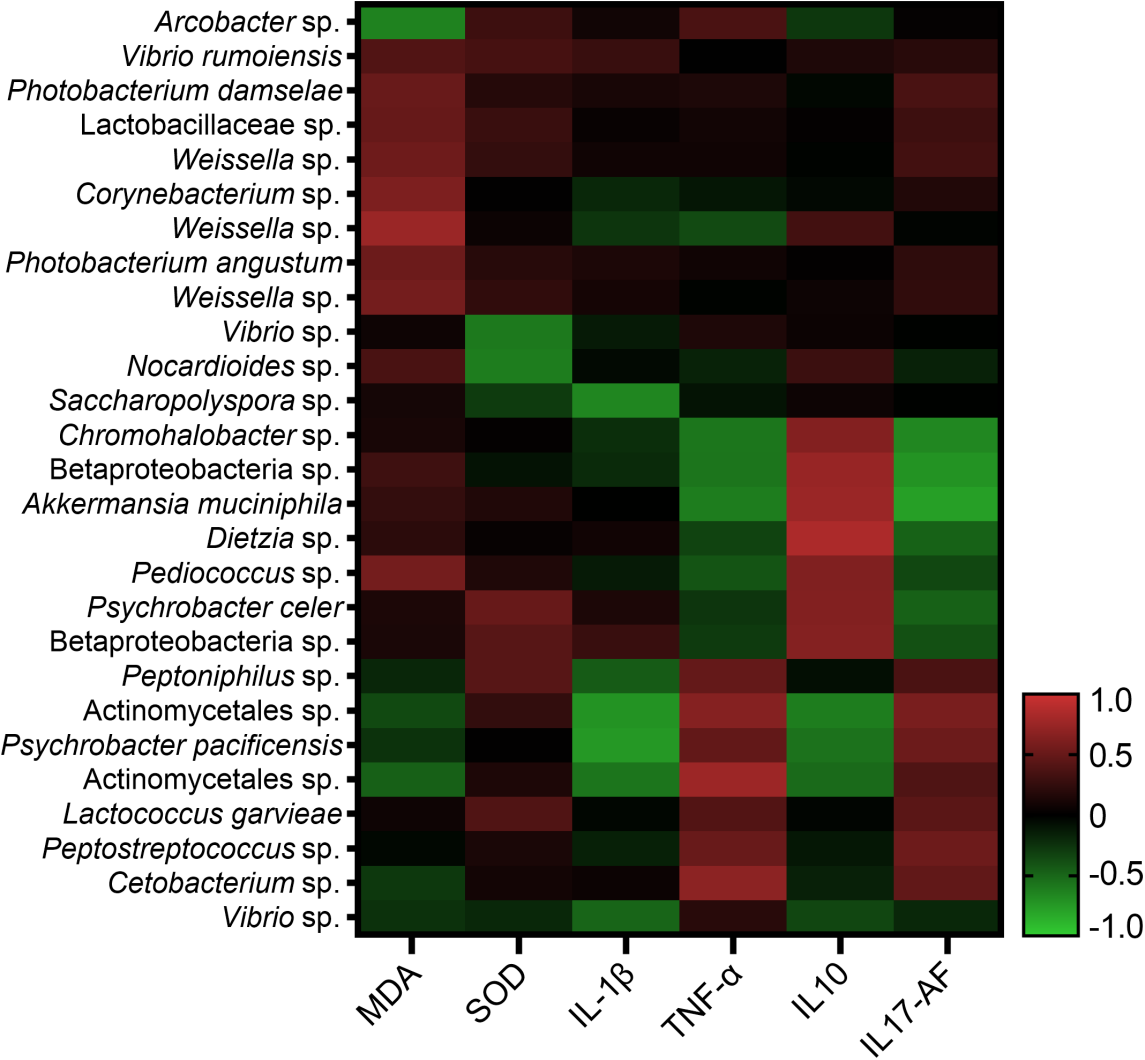
Correlation analysis of 27 ASVs of intestinal bacterial community and immune response parameters.

All ASVs of the intestinal bacterial community were examined in all the 36 samples, and 114 ASVs were identified for the correlation analysis with immune response parameters. Among the 114 ASVs, 27 ASVs had significant correlations with one or more immune response parameters through Spearman’s correlation analysis or Pearson’s correlation analysis. The correlation values between the 27 ASVs and the immune response parameters in Spearman’s correlation analysis and Pearson’s correlation analysis ranged in 0.667-0.832 and 0.666-0.898 respectively (**Figure 7**). *Arcobacter* was found to have significant correlation with MDA content with the correlation value of -0.72 (*P* < 0.05) in Pearson’s correlation analysis, while without significant correlation with MDA content in Spearman’s correlation analysis. A total of seven ASVs were found to have significant correlations with one or more immune response parameters through both Spearman’s correlation analysis and Pearson’s correlation analysis (**Table S5**). They were annotated as *Weissella* sp., *Akkermansia muciniphila*, *Dietzia* sp., *Psychrobacter pacificensis*, Actinomycetales sp., Betaproteobacteria sp. and Actinomycetales sp.

## Discussion

The intestinal bacteria of fish can be broadly divided into autochthonous species that colonized the intestinal mucosa and the allochthonous species that free live and could not attach to the intestinal mucosa (Dehler et al., 2017). The autochthonous bacteria on the intestinal mucosa was obviously different from the allochthonous bacteria in digesta (Feng et al., 2011). The autochthonous bacteria always plays more important roles than allochthonous bacteria, as they could block the attachment sites of pathogens and provide disease prevention support to hosts (Banerjee and Ray, 2017). The allochthonous bacteria in the intestinal tract of pufferfish has been investigated in different development stages (Tanasomwang, 1989) and feeding with specific commercial feed formulations (Su et al., 2017). The present study was focused on the autochthonous bacterial community on pufferfish intestinal mucosa in order to illuminate its characteristics during aquaculture period and the possible relationship with immune response. The predominant phyla in the intestinal bacterial community were Proteobacteria, Firmicutes, Cyanobacteria, Spirochaetes and Tenericutes. Proteobacteria was found to be the most dominant phyla, which accounted for 68.82% of the total bacterial abundance. The result was consistent with previous studies on relative abundance of Proteobacteria in fish intestinal tract with 45.5 ± 18.3% in rainbow trout (Ingerslev et al., 2014) and 59-87% in common carp (Li et al., 2013).

The predominant genera in the intestinal bacterial community were *Arcobacter*, *Photobacterium*, *Vagococcus*, *Weissella* and *Vibrio*, which was different from the previous studies of pufferfish (Talwar et al., 2018; Ou et al., 2019). *Arcobacter*, *Photobacterium* and *Vibrio* contains some common pathogenic species for aquatic species. For example, *Photobacterium damselae* is a neuraminidases producer and infectious agent and is associated with the skin ulcers of fish (Egerton et al., 2018). *Vagococcus fluvialis* was reported to be a good candidate of probiotic as it showed good protection against *Vibrio* challenge (Sorroza et al., 2012). Some species in *Weissella* are proposed as probiotics, whereas other species might be opportunistic pathogens (Mortezaei et al., 2020). The previous studies were focused on the allochthonous bacteria, and *Vibrio*, *Enterobacter*, *Bacillus*, *Bacteroides* and *Escherichia* were reported to be the predominant genera (Li et al., 2015; Su et al., 2017). *Vibrio* was found be the dominant genus in the autochthonous intestinal bacteria in this study and also in the allochthonous intestinal bacteria in other literatures (Li et al., 2015; Yu et al., 2019; Kong et al., 2021). *Vibrio* is one of the most important bacterial genera in aquaculture and occupies the species as opportunistic pathogens causing infections such as *V*. *anguillarum*, *V*. *salmonicida* and *V*. *vulnificus* (Austin et al., 2007), and also has probiotic such as *V*. *alginolyticus* (Austin et al., 1995). It was reported that the intestinal tracts of marine fish are predominantly colonized by the members of the genus *Vibrio* (Shiina et al., 2006). In addition, there were some differences in intestinal bacterial communities between pufferfish and other fish species (e.g., Actinobacteria and Bacteroides usually dominant in other fish species) (Li et al., 2016; Nie et al., 2017), indicating that host phylogeny played important roles in the determination of the composition of fish intestinal bacteria (Xiong et al., 2019).

In the present study, *Arcobacter* was found to be the most core bacterial taxon in the intestinal bacterial community of pufferfish, with the most dominant abundance at the genus level and dominant positions in co-occurrence relationships with other bacterial taxa. *Arcobacter* has been reported to be dominant in the intestinal bacterial community of pufferfish (not specific for autochthonous bacteria) under some conditions, such as excess dietary astaxanthin (ASTX, 500 mg/kg) and arachidonic acid (ARA, 23.41 g/kg) (Ou et al., 2019; Yu et al., 2019). In the present study, *Arcobacter* was first found to be consistently highly enriched during the aquaculture period. *Arcobacter* is always regarded as emergent enteric pathogens and potential zoonotic agents, and it is frequently isolated from ill animals and humans with enteritis (Phillips, 2001; Ou et al., 2019). *Arcobacter* was also reported to be dominant in ulcer mucus surfaces of fish and moribund marine invertebrates (Lokmer and Mathias Wegner, 2015; Karlsen et al., 2017). However, pufferfishes did not show obvious disease symptoms under the high abundance of *Arcobacter* in the present study, indicating that *Arcobacter* might play other roles instead of pathogens in pufferfish. Immune system of mucosal tissues influences pathobionts decisions to be pathogenic or nonpathogenic. *Arcobacter* was also reported to be the dominant genus in the intestinal tract of deep-sea hagfishes (Lian et al., 2021). Phage genome was found to be inserted and integrated into the genome of *Arcobacter* with RM system, multiple TA systems and two CRISPR-Cas systems. It was speculated that *Arcobacter* might have the ability to resist viruses and could help relieve viral pressure for the hosts of hagfishes. *Arcobacter* was thus suggested to play important roles in the intestinal autochthonous microbiota of healthy pufferfish, and further study is needed to clarify the mechanism.

The mean levels of alpha diversity of intestinal bacterial communities in RAS and OSCS systems were very close, but the alpha diversity range in OSCS was more variable than that in RAS. The separation of beta diversity unweighted UniFrac distances between RAS and OSCS samples was probably caused by the differences in aquaculture systems between RAS and OSCS systems, including water environment, developmental stages of pufferfish and dietary composition (Kokou et al., 2019). The significant correlations among the concentrations of nutritive salts and between the concentrations of nutritive salts and aquaculture systems in this study indicated that different aquaculture systems occupied specific water environment qualities, which may affect the intestinal bacterial community. Intestinal bacterial diversity could be used as a biomarker of fish health because lower bacterial diversity was frequently detected in diseased fish (Xiong et al., 2019). The information of bacterial diversity variation in fish intestinal bacterial communities in RAS and OSCS systems is helpful for early warning of intestinal bacteria disorder and disease occurrence.

In regards to the differences of intestinal bacterial communities between RAS and OSCS, the abundances of some common phyla were found to be relatively higher in OSCS than that in RAS such as Spirochaetes and Bacteroidetes, which is consistent with previous studies (Shiina et al., 2006; Dehler et al., 2017; Su et al., 2017). Spirochaetes forms a monophyletic phylum of bacteria and has several genera that contain pathogenic species, which could lead to intestinal spirochetosis (Hampson and Ahmed, 2009). Spirochaetes was commonly detected in the digestive tracts of arthropods and several species of mammals and regularly found deep in marine sediments and soils, but at low concentrations in the intestinal microbiota of fish (Yoshida et al., 2022). The Spirochaetes abundance in the seawater in OSCS system was extremely lower than that in the intestine samples in this study, suggesting that the high abundance of Spirochaetes in the intestine samples in OSCS system was not caused by the passive transfer from seawater but possibly due to the active selection by fish. Bacteroidetes is one of the largest phyla of Gram-negative bacteria inhabiting mammal gastrointestinal tract and is considered a cornerstone of sophisticated homeostasis in fish (Gibiino et al., 2018; Talwar et al., 2018; Wang et al., 2018). The low Bacteroidetes/Firmicutes ratio could lead to obesity in mammals and fast growth of fish (Li et al., 2013). The lower Bacteroidetes/Firmicutes ratio in the intestine samples in RAS compared with that in OSCS in this study may suggest the intestinal bacteria in RAS have more favorable roles in growth promotion than that in OSCS. According to LEfSe analysis, three of the top-4 enriched ASVs of intestinal bacterial communities in RAS belonged to Enterococcaceae, and three of the top-4 enriched ASVs in OSCS belonged to Vibrionaceae. Enterococcaceae performs wide diversity of functions in intestinal microbiota. Enterococcaceae spp. was reported to have an active role in maintaining immunologic homeostasis within the pouch mucosa (Smith et al., 2005; Scarpa et al., 2011), and Enterococcaceae in intestine also functioned as a master regulator of host defense against radiation, protecting both hematopoietic and gastrointestinal systems (Guo et al., 2020). Vibrionaceae occupies some pathogenic species especially in the genus *Vibrio*, and the functional pathways of intestinal bacteria related to *Vibrio* were also found to be more enriched in OSCS than that in RAS in this study. The enriched intestinal bacterial taxa in OSCS, such as Spirochaetes and Vibrionaceae, contain some bacterial species that are pathogenic to aquatic animals, while in most cases they do not cause disease. Pathogenic bacteria could be more prevalent and cause infection and disease only when the balance of intestinal commensal microbiota is disturbed (de Bruijn et al., 2018). It is suggested that the intestinal bacteria in OSCS are more enriched in potentially pathogenic species, and more attention should be paid to that during the aquaculture of pufferfish.

The alpha diversity of the seawater bacterial community was significantly higher than that of the intestinal bacterial community, which was consistent with previous reports (Duan et al., 2020; Kuang et al., 2020). It is reasonable that the water environment provides ideal habitat for various bacterial communities to have high diversity, and intestinal bacteria are constrained by abiotic and biotic factors, such as nutrition, salinity and host immune system (Kuang et al., 2020). However, there were some similarities of bacterial communities between intestinal and seawater samples. For instance, Proteobacteria and Cyanobacteria were found to be dominant phyla in both intestinal and seawater bacterial communities. Nevertheless, no significant correlation was found in the abundance of the bacterial phyla that shared by intestinal tract and seawater samples, and more differences were found in bacterial communities between intestinal and seawater samples, in terms of bacterial community taxa and functions. It is suggested that the changes of bacterial communities in intestinal tract and seawater are independent during the aquaculture period. Compared with terrestrial animals, environmental bacteria has a considerable impact on bacterial compositions of the fish intestinal tract due to constant contact with water (Kuang et al., 2020). Fish select and enrich the intestinal bacteria from the surrounding water during early development (Vadstein et al., 2018), and the environmental bacteria can be transferred from the water environment to the intestinal tract at any time through water ingestion (Kuang et al., 2020). At the same time, a series of exogenous and endogenous factors could affect the colonization process of bacteria in the fish intestinal tract, among which food sources, developmental stage and physiological status are of the most significance. Therefore, intestinal bacteria are not a passive collection of the environmental free-living bacterial communities. Intestinal bacterial community of aquatic species always exhibit stability, and the bacterial community in the hemolymph of Pacific oyster (*Crassostrea gigas*) was stable from June to August based on the survey in Germany and Netherlands (Lokmer et al., 2016). In the present study, the increasing F-values of the beta diversity with the sampling months indicated that the seawater bacteria play important roles in intestinal bacterial colonization during early development, and the differences between the intestinal bacteria and seawater bacteria tended to increase along with the aquaculture process, when the transfer from RAS to OSCS will further increase the differences.

The intestinal bacteria can participate in the host inflammatory response by cytokine-microbiota or microbiota-cytokine modulation interactions and influence the immune homeostasis and stress response (Mendes et al., 2019). From July to August, the pufferfish had been transferred from RAS to OSCS, and MDA contents, SOD activities and the relative expression levels of TNF-α, IL10 and IL17-AF changed significantly, indicating the immune response of pufferfish. On one hand, the pufferfishes had to acclimate to the new environment of the offshore sea cage. The water quality and seawater bacterial composition in OSCS were much more complex than that in RAS, which could induce stress on pufferfish. On the other hand, biotic and abiotic stresses happened more frequently in summer than that in the other seasons. For instance, the abundance of some marine pathogenic bacteria generally increased with the elevated water temperature (Oberbeckmann et al., 2012; Froelich et al., 2015), and the abundance of *Vibrio* was found to reached a peak in summer in the North Yellow Sea, where the present study was conducted (Gao et al., 2022a). The increasing pathogen abundance might raise the stress of immune response and induce acute inflammatory processes and cytokine variations in July and August. In addition, the elevated water temperature and salinity variation caused by rainfall were other factors causing abiotic stress on pufferfish (Liu et al., 2014). Therefore, the immune response of pufferfish should be monitored more strictly during the aquaculture period from July to August, to ensure the safety of aquaculture.

In the present study, four bacterial taxa at the genus level, *Weissella* sp., *Akkermansia muciniphila*, *Dietzia* sp. and *Psychrobacter pacificensis*, were found to have significant correlations with one or more immune response parameters through both Spearman’s correlation analysis and Pearson’s correlation analysis. The species in *Weissella* and *Dietzia* include both probiotics and opportunistic pathogens (Click, 2011; Mortezaei et al., 2020; Brown et al., 2022). *Akkermansia* spp. are widely present in the intestinal tract of human and animals and are negatively associated with metabolic disorder in clinical studies (Derrien et al., 2017; Parolini, 2019). The knowledge of the function of *Psychrobacter pacificensis*, which was first isolated from deep seawater, in intestinal microbiota is very limited, while it was speculated to be evolved from a pathobiont (Welter et al., 2021). The maintenance of health status of fish always relies on the balance between the immune system and endogenous bacteria, especially the autochthonous bacteria (Gómez and Balcázar, 2008). The intestinal bacteria is able to modulate the host immune system by regulating expressions of immunity-related genes in the hosts and producing various substances, such as antimicrobial compounds and sebastenoic acid (Rawls et al., 2007). In addition, the colonizing of autochthonous bacteria provides colonization resistance to other bacteria introduced later and prevents the invasion of potentially pathogenic bacteria. Recently, the specific bacteria taxa in the shrimp intestinal tract were reported as indicators of shrimp health and disease (Xiong et al., 2015; Huang et al., 2018). The correlations between intestinal autochthonous bacteria taxa and immune response parameters in spleen were analyzed in this study to identify the bacteria taxa that may be related to immune response. However, there are some other important immune organs that were not included in the immune response analysis of the present study, such as head kidney, as well as intestinal tract, which is the first barrier between host and external environment. It should be noted that further analysis on the immune response parameters in other immune organs will be necessary to fully understand the mechanism of how intestinal autochthonous bacteria modulate immune response. Overall, in this study, the above four bacterial taxa were suggested to be the indicators for immune status and pathological process of pufferfish.

## Conclusion

The characteristics of both intestinal autochthonous bacteria of pufferfish and seawater bacteria were analyzed during the aquaculture period in RAS and OSCS. *Arcobacter* was found to be the core bacterial taxon in the intestinal autochthonous bacteria of pufferfish during the aquaculture period. Increasing differences were found between the intestinal and seawater bacterial communities along with the aquaculture process. Four bacterial taxa had significant correlations with immune response parameters, and they were suggested to be the indicators for immune status and pathological process of pufferfish. The results will contribute to the understanding of intestinal bacterial homeostasis and biosecurity practice in pufferfish aquaculture.

## Data availability statement

The datasets presented in this study can be found in online repositories. The names of the repository/repositories and accession number(s) can be found below: https://www.ncbi.nlm.nih.gov/genbank/, PRJNA884649.

## Ethics statement

The animal study was reviewed and approved by ethics committee of Dalian Ocean University.

## Author contributions

LG, investigation, sample collection, methodology, formal analysis, and writing original draft. ZZ, ZX, and QL, investigation and sample collection. NK, sample collection. LW, writing review and editing, supervision, and funding acquisition. LS, writing review and editing, supervision, and funding acquisition. All authors contributed to the article and approved the submitted version.
