# Supplementary material for: The variation of intestinal autochthonous bacteria in cultured tiger pufferfish Takifugu rubripes
Source: Front Cell Infect Microbiol. 2022 Dec 13;12:1062512. doi: 10.3389/fcimb.2022.1062512 (PMC9792791; doi:10.3389/fcimb.2022.1062512)
Supplement: Supplementary file 1 [file Table_1.docx]

**TABLE** **S1**

Average correlation values of the results of Spearman’s correlation analysis and Pearson’s correlation analysis between water quality indexes or between water quality index and aquaculture system (RAS/OSCS).

|  | T | DO | pH | Sal | TAN | NO_2_-N | NO_3_-N | PO_4_-P |
| --- | --- | --- | --- | --- | --- | --- | --- | --- |
| DO | -0.1485 |  |  |  |  |  |  |  |
| pH | -0.0545 | -0.069 |  |  |  |  |  |  |
| Sal | -0.498 | -0.1835 | -0.2255 |  |  |  |  |  |
| TAN | -0.376 | -0.592 | 0.2095 | 0.1755 |  |  |  |  |
| NO_2_-N | -0.427 | -0.3995 | -0.121 | 0.1735 | **0.6995*** |  |  |  |
| NO_3_-N | -0.2455 | -0.557 | 0.0965 | -0.0725 | **0.6135*** | 0.5805 |  |  |
| PO_4_-P | -0.275 | -0.6575 | 0.201 | 0.0485 | **0.78*** | **0.6705*** | **0.9075*** |  |
| RAS/OSCS | 0.3765 | 0.5645 | 0.0175 | -0.0705 | **-0.8105*** | **-0.625*** | **-0.8345*** | **-0.811*** |

Note: The values with significant correlation (*P* < 0.05) in both Spearman’s correlation analysis and Pearson’s correlation analysis are flagged with ‘*’ and bolded.

**TABLE** **S2**

Average correlation values of the results of Spearman’s correlation analysis and Pearson’s correlation analysis between water quality index and the beta diversity of intestinal bacterial community.

|  | Beta diversity |
| --- | --- |
| T | 0.0535 |
| DO | 0.5045 |
| pH | -0.525 |
| Sal | 0.2995 |
| TAN | -0.759 |
| NO_2_-N | -0.6275 |
| NO_3_-N | -0.39 |
| PO_4_-P | -0.53 |

Note: No value with significant correlation (*P* < 0.05) in both Spearman’s correlation analysis and Pearson’s correlation analysis is found.

**TABLE** **S3**

Average correlation values of the results of Spearman’s correlation analysis and Pearson’s correlation analysis between the abundance of the shared bacterial phyla in intestinal tract and seawater samples.

| Phylum | Correlation value | Phylum | Correlation value |
| --- | --- | --- | --- |
| Acidobacteria | -0.0765 | Fusobacteria | 0.037 |
| Actinobacteria | 0.0145 | Gemmatimonadetes | 0.083 |
| Armatimonadetes | 0.0025 | Lentisphaerae | -0.1415 |
| Bacteroidetes | -0.297 | Nitrospirae | 0.0215 |
| Chlamydiae | -0.02 | Planctomycetes | -0.162 |
| Chloroflexi | 0.188 | Proteobacteria | -0.323 |
| Cyanobacteria | -0.241 | Spirochaetes | 0.104 |
| Deferribacteres | 0.184 | TM7 | -0.0475 |
| Elusimicrobia | 0.097 | Tenericutes | 0.097 |
| Fibrobacteres | 0.126 | Verrucomicrobia | -0.003 |
| Firmicutes | 0.0855 | Thermi | -0.0865 |

Note: No value with significant correlation (*P* < 0.05) in both Spearman’s correlation analysis and Pearson’s correlation analysis is found.

**TABLE** **S4**

Average correlation values of the results of Spearman’s correlation analysis and Pearson’s correlation analysis between water quality index and immune response parameter.

|  | MDA | SOD | IL-1β | TNF-α | IL10 | IL17-AF |
| --- | --- | --- | --- | --- | --- | --- |
| T | 0.567 | -0.221 | -0.1565 | 0.042 | -0.0675 | -0.008 |
| DO | -0.0355 | 0.0035 | 0.444 | -0.181 | 0.2625 | 0.1415 |
| pH | -0.4435 | 0.374 | -0.4995 | 0.685 | -0.4935 | 0.5315 |
| Sal | -0.1925 | -0.256 | 0.22 | -0.5455 | 0.5215 | -0.6465 |
| TAN | -0.1265 | 0.161 | -0.699 | 0.18 | -0.4015 | -0.1005 |
| NO_2_-N | -0.2435 | 0.482 | -0.461 | 0.0805 | -0.2945 | -0.1335 |
| NO_3_-N | -0.439 | 0.5845 | -0.673 | 0.441 | -0.402 | 0.009 |
| PO_4_-P | -0.4415 | 0.377 | -0.6615 | 0.389 | -0.421 | -0.0855 |

Note: No value with significant correlation (*P* < 0.05) in both Spearman’s correlation analysis and Pearson’s correlation analysis is found.

**TABLE** **S5**

Average correlation values of the results of Spearman’s correlation analysis and Pearson’s correlation analysis between 27 ASVs of intestinal bacterial community and immune response parameters.

|  | MDA | SOD | IL-1β | TNF-α | IL10 | IL17-AF |
| --- | --- | --- | --- | --- | --- | --- |
| *Arcobacter* sp. | -0.635 | 0.293 | 0.0815 | 0.3595 | -0.272 | 0.021 |
| *Vibrio rumoiensis* | 0.392 | 0.3395 | 0.272 | 0.0045 | 0.1455 | 0.192 |
| *Photobacterium damselae* | 0.507 | 0.1765 | 0.116 | 0.1405 | -0.0355 | 0.356 |
| Lactobacillaceae sp. | 0.497 | 0.2745 | 0.0355 | 0.083 | 0.015 | 0.294 |
| *Weissella* sp. | 0.5325 | 0.2475 | 0.0745 | 0.075 | -0.0185 | 0.318 |
| *Corynebacterium* sp. | 0.608 | 0.007 | -0.1905 | -0.112 | -0.0415 | 0.159 |
| *Weissella* sp. | **0.747*** | 0.058 | -0.2615 | -0.3655 | 0.32 | -0.024 |
| *Photobacterium angustum* | 0.524 | 0.186 | 0.136 | 0.075 | 0.0135 | 0.223 |
| *Weissella* sp. | 0.5615 | 0.23 | 0.0995 | -0.018 | 0.061 | 0.234 |
| *Vibrio* sp. | 0.0665 | -0.5935 | -0.1285 | 0.1455 | 0.0585 | -0.014 |
| *Nocardioides* sp. | 0.3555 | -0.6115 | -0.0445 | -0.1685 | 0.285 | -0.1645 |
| *Saccharopolyspora* sp. | 0.101 | -0.291 | -0.655 | -0.095 | 0.0605 | -0.0115 |
| *Chromohalobacter* sp. | 0.1145 | 0.0165 | -0.226 | -0.5795 | 0.6365 | -0.661 |
| Betaproteobacteria sp. | 0.301 | -0.092 | -0.209 | -0.5755 | 0.7325 | **-0.719*** |
| *Akkermansia muciniphila* | 0.2485 | 0.156 | -0.008 | -0.61 | **0.751*** | **-0.777*** |
| *Dietzia* sp. | 0.2055 | 0.0295 | 0.0785 | -0.3315 | **0.835*** | -0.479 |
| *Pediococcus* sp. | 0.561 | 0.1485 | -0.127 | -0.4115 | 0.6235 | -0.347 |
| *Psychrobacter celer* | 0.1355 | 0.5 | 0.1325 | -0.26 | 0.63 | -0.476 |
| Betaproteobacteria sp. | 0.1235 | 0.424 | 0.27 | -0.284 | 0.641 | -0.391 |
| *Peptoniphilus* sp. | -0.186 | 0.4225 | -0.4485 | 0.4855 | -0.0725 | 0.36 |
| Actinomycetales sp. | -0.359 | 0.2435 | **-0.719*** | 0.644 | -0.6135 | 0.5875 |
| *Psychrobacter pacificensis* | -0.2455 | 0.008 | **-0.7415*** | 0.4765 | -0.5605 | 0.522 |
| Actinomycetales sp. | -0.4715 | 0.1375 | -0.574 | **0.7595*** | -0.5205 | 0.3825 |
| *Lactococcus garvieae* | 0.0675 | 0.3895 | -0.0325 | 0.4 | -0.028 | 0.4365 |
| *Peptostreptococcus* sp. | -0.0375 | 0.124 | -0.1565 | 0.5025 | -0.116 | 0.528 |
| *Cetobacterium* sp. | -0.276 | 0.089 | 0.055 | 0.6805 | -0.1605 | 0.473 |
| *Vibrio* sp. | -0.242 | -0.1905 | -0.494 | 0.1925 | -0.344 | -0.1915 |

Note: The values with significant correlation (*P* < 0.05) in both Spearman’s correlation analysis and Pearson’s correlation analysis are flagged with ‘*’ and bolded.
